# Longitudinal dynamics of depression in risk groups of older individuals during the COVID-19 pandemic

**DOI:** 10.3389/fepid.2023.1093780

**Published:** 2023-02-13

**Authors:** Theresa Dankowski, Lydia Kastner, Ulrike Suenkel, Anna-Katharina von Thaler, Christian Mychajliw, Michael Krawczak, Walter Maetzler, Daniela Berg, Kathrin Brockmann, Ansgar Thiel, Gerhard W. Eschweiler, Sebastian Heinzel

**Affiliations:** ^1^ Department of Neurology, Kiel University, University Medical Centre Schleswig-Holstein, Kiel, Germany; ^2^ Institute of Medical Informatics and Statistics, Kiel University, University Medical Centre Schleswig-Holstein, Kiel, Germany; ^3^ Institute of Sport Science, Eberhard Karls University of Tübingen, Tübingen, Germany; ^4^ Department of Psychiatry and Psychotherapy, Tübingen University Hospital, Tübingen, Germany; ^5^ Lead Graduate School and Research Network, University of Tübingen, Tübingen, Germany; ^6^ Department of Neurodegeneration, Hertie Institute for Clinical Brain Research, University of Tübingen, Tübingen, Germany; ^7^ German Center for Neurodegenerative Diseases, University of Tübingen, Tübingen, Germany; ^8^ Geriatric Center, Tübingen University Hospital, Tübingen, Germany

**Keywords:** depression, stress, resilience, psychosocial factors, pandemic, corona, older population, COVID-19

## Abstract

**Background:**

Older individuals are most at risk of severe COVID-19 and particularly require protection causing (self)restriction of psychosocial interaction in daily living. So far, the impact of psychosocial withdrawal on mental health seems less pronounced in community-dwelling older individuals compared to younger individuals. However, dynamics and adverse long-term effects of the pandemic, such as increases in depression, are still mostly unclear, especially for vulnerable subgroups.

**Methods:**

Pre-pandemic and 3-, 8-, 14-, 20-month peri-pandemic data were analyzed in 877 older participants (age at 3-month peri-pandemic: mean ± SD: 72.3 ± 6.3, range: 58–91 years) of the observational prospective TREND study in Germany. Severity of depression (Beck's Depression Inventory-II scores) and key factors of (mental) health were investigated for cross-sectional associations using path modeling. Risk groups defined by resilience, loneliness, history of depression, stress, health status and fear of COVID-19 were investigated for differences in depression between timepoints.

**Findings:**

The early pandemic (3-month) severity of depression was most strongly associated with history of depression, stress and resilience. Overall increases in clinically relevant depression (mild-severe) from pre- to 3-month peri-pandemic were small (% with depression at pre-/3-month peri-pandemic: 8.3%/11.5%). Changes were most pronounced in risk groups with low resilience (27.2%/41.8%), loneliness (19.0%/28.9%), fear of COVID-19 (17.6%/31.4%), high stress (24.4%/34.2%), a history of depression (27.7%/36.9%), and low health status (21.8%/31.4%). Changes in depression were largely observed from pre- to 3-month and were sustained to the 20-month peri-pandemic timepoint, overall and in stratified risk groups defined by single and cumulative risk factors. Changes between timepoints were heterogenous as indicated by alluvial diagrams.

**Conclusion:**

Only specific risk groups of older individuals showed a large increase in depression during the COVID-19 pandemic. Since these increases occurred early in the pandemic and were sustained over 20 months, these vulnerable risk groups need to be prioritized for counselling and risk mitigation of depression.

## 1. Introduction

While advanced age proved to be a major risk factor for severe adverse health effects and mortality during the Corona Virus Disease 2019 (COVID-19) pandemic (1–3) , public health measures and precautionary behavior protected many at risk against SARS-CoV-2 infection and severe COVID-19 (4). However, social isolation and (self-)restriction of psychosocial interaction in everyday life may contribute to indirect consequences of the pandemic, such as perceived distress and symptoms of depression and/or anxiety (5–7) . Surprisingly, current evidence suggests that community-dwelling older citizens appear to be less psychosocially affected by the COVID-19 pandemic and related restrictions in comparison to young and mid-age individuals (8–10). Increases in moderate to severe depressive symptoms and perceived stress, especially at the beginning of the COVID-19 pandemic, were mainly observed in participants younger than 60 years of age (9, 11). Therefore, many older individuals are hypothesized to have greater resilience and to follow adaptive coping strategies to preserve well-being during the COVID-19 pandemic (6, 9). Resilience as the capacity to recover quickly from stressful events and challenges is not only a personality trait but can be considered a dynamic process allowing positive adaptation in a context of significant adversity such as the COVID-19 pandemic (12, 13). Thus, resilience may be pivotal for preventing both depressive symptoms in the beginning as well as the development of long-term depression, which often has severe personal and clinical relevance (14). Conversely, female gender, higher levels of loneliness and living alone, physical inactivity, long-standing illness and younger age have been shown to impose a higher risk for increased depressive symptoms during the first year of the COVID-19 pandemic (15, 16).

However, pandemic-related changes in mental health have been reported to be highly heterogeneous among individuals and subgroups (14, 17) possibly indicating diverse constellations of risk and protective factors. Moreover, the COVID-19 pandemic is continually characterized by phases of exponential growth rates of infections, unanticipated developments, and multifacetedness that determine public health policy making as well as everyday lives, livelihoods, worries, challenges, and perspectives of individuals. Consequently, time-lag effects and heterogenous longitudinal dynamics of changes in the severity of depression and other mental health problems may be observed, especially in at-risk subgroups. However, in older individuals the dynamic changes in the (clinically relevant) severity of depression from pre-pandemic to early pandemic to longitudinal peri-pandemic phases of the COVID-19 pandemic are still unclear and subgroups with potentially increased vulnerability have not been delineated, yet.

The present study therefore aimed to 1) assess pre- to peri-pandemic changes in the severity of depression and key psychosocial and pandemic-related factors of mental health in a large, widely phenotyped cohort of older individuals, 2) identify risk factors related to increased clinically relevant depression in the early pandemic, and 3) investigate risk group-specific changes pre- to 3-month peri-pandemic as well as longitudinal changes in the severity of depression.

## 2. Materials and methods

### 2.1. Study population and peri-pandemic surveys

In the present study, we analyzed data from the prospective Tübingen Evaluation of Risk Factors for Early Detection of Neurodegeneration (TREND) study (www.trend-studie.de/english). Initiated in 2009, 1,201 older individuals from the Neckar-Alb and Stuttgart area in Germany were recruited from the general population and have been enriched by individuals with established risk factors for the development of Alzheimer's and Parkinson's disease, including life-time depression, olfactory loss, and/or possible REM-sleep behavior disorder. The TREND cohort has been studied longitudinally in six waves from 04/2009 to 03/2020 every two years by multimodal and multidisciplinary data acquisition (*pre-pandemic* data).

**Figure 1:**
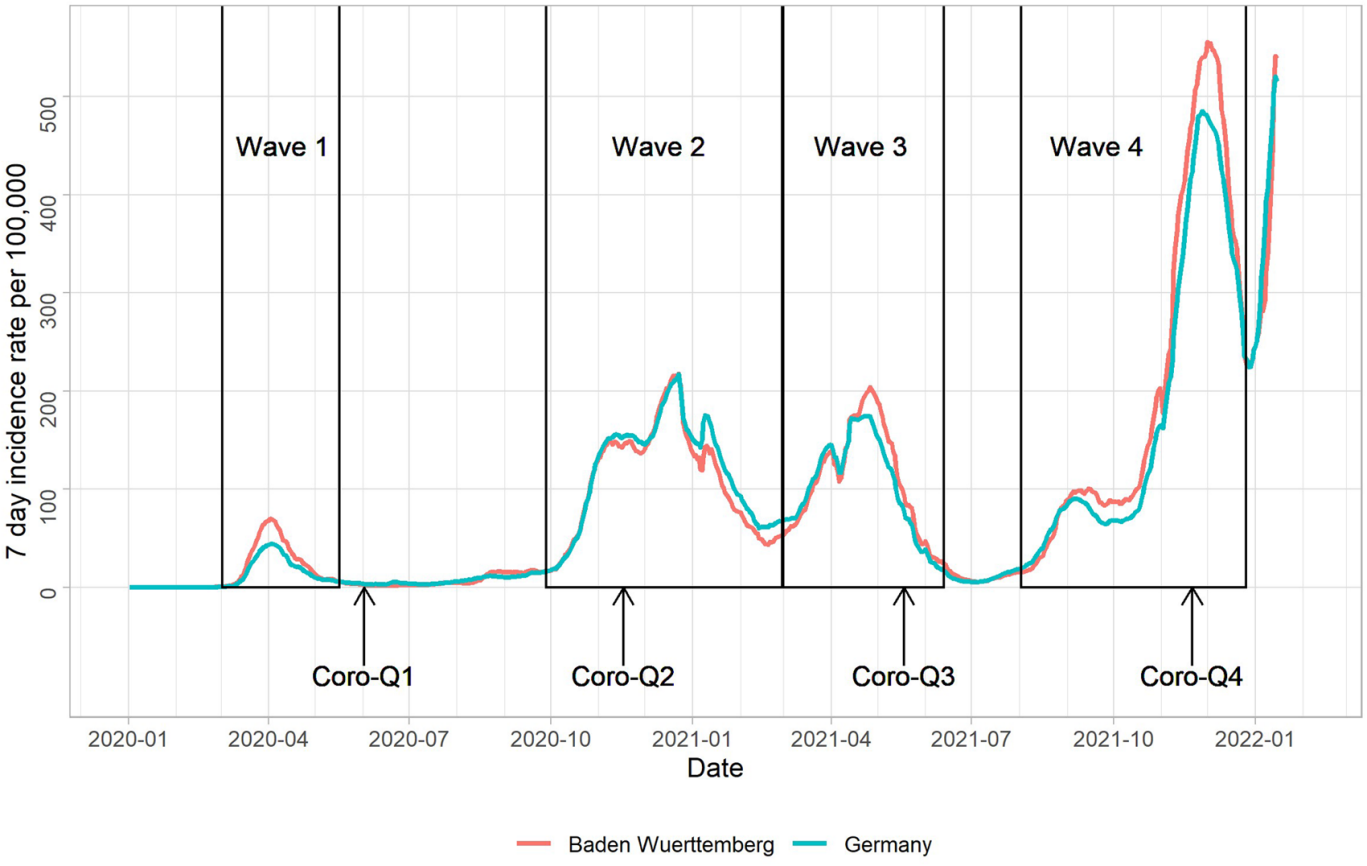
Timing of Coro-Q surveys and 7-day incidence rates of SARS-CoV2 infections in Germany and the state of Baden-Württemberg (residence area of TREND participants) as reported by the Robert-Koch-Institute.

At the onset of the COVID-19 pandemic, some 900 individuals still actively participated in the TREND study. Postal surveys (Coro-Q) were sent to these participants comprising custom and established, validated questionnaires on generic, psychosocial, health- and pandemic-related aspects (*peri-pandemic* data). The first survey (Coro-Q1) was sent by mail in May 2020 and subsequent Coro-Qs were sent about every six months either as mail or online questionnaires, depending on the participants preference. The response rates for each of the Coro-Qs was >80%. Peri-pandemic data of postal surveys considering an onset of the first COVID-19 pandemic wave in Germany (18) on March 2nd 2020 were at 3-month (Coro-Q1, date of investigation [median (interquartile range; IQR)]: 02/06/2020 (9 days)), 8-month (Coro-Q2, 17/11/2020 (7 days)), 14-month (Coro-Q3, 18/05/2021 (6 days)) and 20-month (Coro-Q4, 21/11/2021 (9 days)). Pre-pandemic data were restricted to observations between 01/01/2017 and 01/03/2020 in the analysis and we used the latest available measurement for all variables. Figure 1 illustrates the timing of Coro-Q surveys and waves of increased SARS-CoV2 infection incidence during the COVID-19 pandemic. Characteristics of the Coro-Qs timing and key demographics of respondents are shown in Table 1.

**Table 1:** Peri-pandemic timing of survey waves and demographic characteristics of respondents. .

Survey wave		3-month Coro-Q1	8-month Coro-Q2	14-month Coro-Q3	20-month Coro-Q4
Response date (median [month/year])		06/2020	11/2020	05/2021	11/2021
Number of surveys sent	Total (*n*)	932	909	899	880
	Paper (*n*, %)	932 (100.0)	909 (100.0)	540 (60.1)	519 (59.0)
	Online (*n*, %)	0 (0.0)	0 (0.0)	359 (39.9)	361 (41.0)
Number of respondents	Total (*n*, %)	774 (83.0)	780 (85.8)	796 (88.5)	754 (85.7)
	Paper (*n*, %)	774 (83.0)	780 (85.8)	445 (82.4)	407 (78.4)
	Online (*n*, %)	–	–	351 (97.8)	347 (96.1)
Age (yrs)	*M*	72.3	72.5	73.0	73.4
	*SD*	6.3	6.3	6.2	6.3
	Range	58–91	59–91	59–92	60–92
Females	*n* (%)	367 (47.4)	377 (48.3)	380 (47.7)	360 (47.7)
Education (yrs)	Median	14.5	14.5	14.5	14.6
	IQR	12–16	12–16	12–16	13–16
	Range	9–21	9–21	9–21	9–22

IQR, interquartile range; *M*, Mean; *SD*, standard deviation; yrs, years.

### 2.2. Study variables

#### 2.2.1. Depression

To assess the severity of depression, we used the Beck's Depression Inventory-II (BDI-II) (19). The BDI-II is a self-report instrument for assessing the severity of 21 symptoms of depression. Participants should select one of four statements which best describes how they have felt in the past two weeks. As a result, 0–13 scores indicate minimal depression, 14–19 mild depression, 20–28 moderate depression and 29–63 severe depression (19). We investigated depression on two different scales: 1) as a quantitative variable (the BDI-II score) measuring the *severity of depression*, and 2) as a categorical variable indicating *clinically relevant depression* (mild, moderate or severe) or non-relevant depression (minimal).

A positive pre-pandemic history of depression was determined based on a self-reported medical lifetime diagnosis of depression at the last onsite TREND visit or BDI-II scores ≥14 in at least one TREND assessment before 01/03/2020.

#### 2.2.2. General health status

Longstanding illness has been associated with longitudinal increases in depression from pre- to early pandemic timepoints (16), and similarly low general health may constitute a risk factor of peri-pandemic changes of depression. To measure participants' general health status, a horizontal visual analog self-report scale (as part of the EQ-5D-5l) was used with endpoints labeled ‘The best health you can imagine” (100 scores) and ‘The worst health you can imagine’ (0 scores) (20).

#### 2.2.3. Physical activity

Since there is a strong negative association between depression and physical activity (21), we decided to analyze physical activity using an ordinal variable with values ‘*no activity’*, ‘*< 1 h (hrs)/week’*, ‘*1–2 h/week’*, ‘*2–4 h/week*’, and ‘*> 4 h of physical activity per week’* with increased heart-rate or sweating using a standardized questionnaire from the German Health survey (22).

#### 2.2.4. Loneliness

Loneliness is strongly associated with depression (23). Therefore, we measured overall loneliness using a 6-item questionnaire (24). Participants were asked to indicate how much each statement applied to them personally (*not at all true, rather not true* or *rather true*, *exactly true)* in the last three months. Indication of (a tendency towards) loneliness was counted for each statement resulting in a total score of 0 to 6 (example item: ‘I miss people who make me feel good’).

#### 2.2.5. Other psychosocial variables

Data on several additional psychosocial factors, such as stress, resilience, and pandemic-related news consumption were also collected. Stress was assessed using the Perceived Stress Scale (25) consisting of 10 items in which participants are asked to indicate how often they felt stressed during the last month (example item: ‘In the last month, how often have you been upset because something unexpected happened?’, with answering options: never, almost never, sometimes, quite often, very often). To measure resilience, we used the Brief Resilience Scale (BRS) consisting of 6 items, e.g., ‘I tend to recover quickly after difficult times’ (26).

Age and gender were also considered as additional variables in the present study, since many empirical studies have shown that these are significant factors influencing depression (27, 28). In addition, we also considered the years of education, a custom question on the ‘*Daily corona pandemic-related news consumption’* (scores: (0) ‘*No news consumption’*, (1) ‘*< 1 h/day’*, (2) ‘*1–2 h/day’*, (3) ‘*2–4 h/day’*, (4) ‘*> 4 hrs’* of pandemic-related news consumption per day) as a potential determinant of depression. Fear of COVID-19 was indicated on a scale from 0 (*not afraid at all*) to 10 (*very much afraid*). Details on the number of observations available for analysis (Supplementary Table S1) and variable definitions (Supplementary Table S2) are provided as Supplementary Material.

### 2.3. Analysis plan and statistical methods

We pursued a stepwise analysis approach to identify subgroups of older individuals with pronounced changes in the (clinically relevant) severity of depression and to investigate overall and subgroup-specific temporal dynamics of depression and their key factors during the COVID-19 pandemic:

1) We visualized and tested pre- to peri-pandemic changes in the severity of depression, loneliness, general health status and physical activity. Differences in continuous as well as categorized variables between five time-points (pre-pandemic and 3-, 8-, 14-, 20-month peri-pandemic timepoints) were assessed for statistical significance using the Wilcoxon signed rank test for paired samples. The overall significance level of α=0.05 with a conservative Bonferroni correction for multiple testing of four longitudinal variables with nine timepoint comparisons, i.e., each individual hypothesis was tested at $\alpha =\frac{0.05}{36}=0.00139$. The number of observations for all considered variables is shown in Supplementary Material (Supplementary Table S1). The longitudinal flow of categorized variables was visualized using alluvial diagrams.
2) To identify potential risk factors of depression and elucidate their interrelations, we investigated which factors were associated with the severity of depression (BDI-II score) at the 3-month peri-pandemic timepoint. A path model was fitted using cross-sectional 3-month peri-pandemic data of the severity of depression as a dependent variable. Covariates of the path models included history of depression, and cross-sectional 3-month peri-pandemic data on perceived stress, loneliness, fear of COVID-19, general health status, physical activity, corona news consumption, education, gender, and age. Resilience was not assessed at the 3-month peri-pandemic timepoint, but at the 8-, 14- and 20-month peri-pandemic timepoints. Therefore, we used the intraindividual average of BRS scores at these timepoints for the path model and the peri-pandemic subgroup stratification. The goodness-of-fit of the path model was improved in an iterative process informed by modification indices given by the software, significance (*p* ≤ 0.05) of path coefficients as well as assumptions on plausible (directional) relationships between the variables. The path model was estimated using robust maximum likelihood and full information maximum likelihood for handling missing values (number of observations used: $n_{used}=740$). Confidence intervals for total effects were estimated using bootstrapping with 1,000 bootstrap samples.
3) Significant associations of potential risk factors of depression with the severity of depression as investigated in the path model, however, might not be valid for ranges of clinically relevant severity of depression and changes thereof from pre- to peri-pandemic timepoints. To identify risk factors of pandemic-related changes of depression, we therefore selected significant path model variables (from analysis step 2) and dichotomized each of these variables to indicate potential subgroups of increased risk of depression. We then determined for each subgroup separately the prevalence of clinically relevant severity of depression (mild, moderate or severe) at the pre- and 3-month peri-pandemic timepoint. Factors defining subgroups with the most pronounced increase in prevalence from pre- and 3-month peri-pandemic were selected as risk factors of depression. The cumulative number of these risk factors (none, one to two, three, and four to six risk factors) as well as low resilience plus one additional risk factor were jointly used for subgroup stratification and further analysis of differential long-term and longitudinal peri-pandemic dynamics of depression in these subgroups. Pairwise comparisons between timepoints were conducted as described above. We considered an overall significance level of α=0.05 with the same Bonferroni correction for multiple testing as described above. Alluvial flow diagrams were used to visualize the subgroup-specific dynamic changes of the (clinically relevant) severity of depression during the COVID-19 pandemic.

Study data were collected and managed using REDCap electronic data capture tools hosted at the University of Tuebingen (29). All statistical analyses were performed with software R version 4.2.1 (30). For path modeling, we used R package *lavaan* version 0.6–12 (31).

## 3. Results

### 3.1. Depression, loneliness, health status and physical activity from pre-pandemic to 3-month peri-pandemic

**Figure 2:**
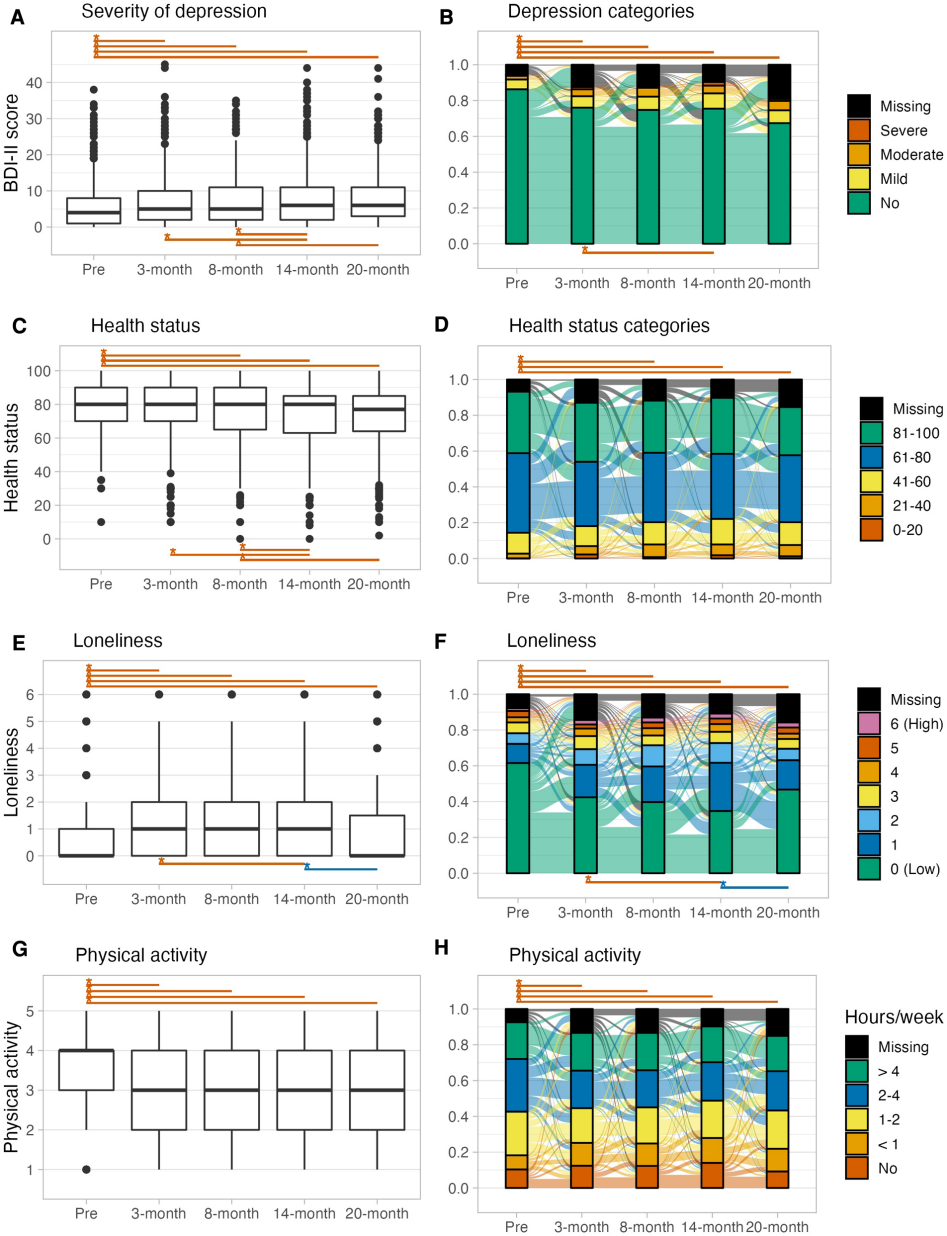
Boxplots (**A,C,E,G**) and alluvial diagrams (**B,D,F,H**) for pre-pandemic and 3-, 8-,14-, 20-month peri-pandemic assessments of the (clinically relevant) severity of depression, loneliness, health status and physical activity. Asterisks and lines indicate significant (*p* < 0.00139, Bonferroni corrected) differences between timepoints (red: deterioration, blue: improvement). Tests for depression and health status in the alluvial diagram are based on categorized data as shown in the plot.

Overall, the severity of depression and loneliness showed a significant increase, and health status and physical activity a significant decrease from pre-pandemic to each peri-pandemic timepoints, except for health status between pre- and 3-month peri-pandemic data (Figure 2, Supplementary Tables S3 and S4). Between peri-pandemic time-points the severity of depression slightly increased and health status slightly decreased further, especially between 8-month and 14-month peri-pandemic. Overall, proportions of individuals with mild to severe depression largely did not change until the 20-month peri-pandemic timepoint (Figure 2B). However, substantial heterogeneity of flow between categories across subsequent timepoints was observed. For example, one participant in the moderate depression severity category at pre-pandemic showed a decrease to mild depression at 3-month peri-pandemic followed by a deterioration to severe depression (8-month), followed by a decline to moderate depression (14-month) and again an increase to severe depression at 20-month peri-pandemic. Proportions of categorized general health and physical activity largely did not change at the group level between 3-months and 20-month peri-pandemic. Loneliness slightly increased between 3- and 14-month peri-pandemic and decreased between 14- and 20-month peri-pandemic. However, these changes were mainly driven by a subgroup with low loneliness. For details on the statistical results and intra-individual differences observed, see Supplementary Material.

### 3.2. Factors associated with the severity of depression at 3-month peri-pandemic

**Figure 3:**
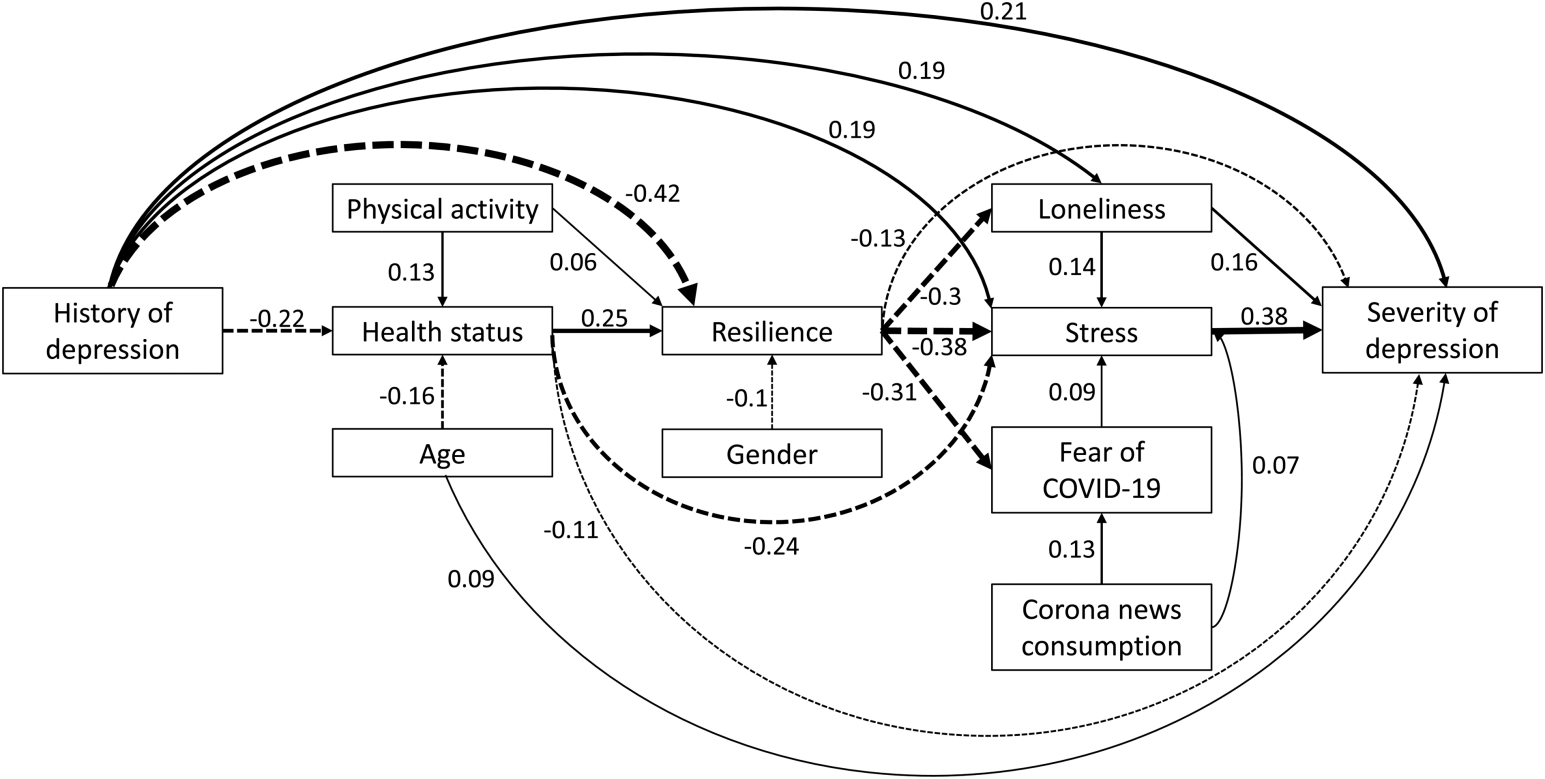
Path model of the severity of depression and relevant factors at the 3-month peri-pandemic timepoint. Numbers indicate standardized regression coefficients of the path model. Solid lines indicate positive effects and dashed lines negative effects. Line widths reflect effect sizes.

A path model was fitted to cross-sectional 3-month peri-pandemic data on the severity of depression and pre-selected putative psychosocial and health-related risk factors for depression. The path model showed a good fit to the data as indicated by a non-significant *χ*2 test (*χ*2 (22) = 31.5, *p* = 0.086) and relevant fit indices (comparative fit index (CFI) = 0.995, Tucker-Lewis index (TLI) = 0.989, root mean square error of approximation (RMSEA) = 0.025 (0.000–0.043), standard root mean squared residual (SRMR) = 0.022). The strength and proposed direction of the observed associations with the severity of depression and/or among factors is shown in Figure 3. Total effects of the associations of factors with the severity of depression are given in Table 2. The strongest associations according to total effects were observed for history of depression, perceived stress, resilience, health status and loneliness. Physical activity, gender, fear of COVID-19, and corona news consumptions showed significant yet only small associations with the severity of depression in the path model. Education did not show any significant association and was therefore excluded from the model.

**Table 2:** Total effects of putative risk factors for increased severity of depression in the path model at 3-month peri-pandemic, and absolute and relative frequencies of individuals with and without these risk factors pre-pandemic and at 3-month peri-pandemic. Risk factors were measured at 3-month peri-pandemic.

Variable	Total effect (95% confidence interval)	Standardized total effect	Definition of risk group	Name of risk group	Size of risk group pre/3-month [*n* (%)]
Depression history (D)	7.79 (6.58, 8.79)	0.55	D = 1	D+	235 (32.2%)/249 (32.4%)
Stress (S)	0.39 (0.3, 0.49)	0.37	S ≥ 14	S+	238 (32.9)/260 (34.0)
Resilience (R)	−3.12 (-3.87, -2.38)	-0.34	R < 3.00	R-	125 (17.8)/134 (18.1)
Health status (HS)	-0.11 (-0.13, -0.08)	-0.29	HS ≤ 60	HS-	142 (19.7)/156 (20.6)
Loneliness (L)	0.82 (0.52, 1.15)	0.20	L ≥ 2	L+	205 (28.9)/218 (29.2)
Age (A)	0.14 (0.08, 0.2)	0.13	A ≥ 75	A+	260 (35.6)/278 (36.2)
Physical activity (PA)	-0.28 (-0.47, -0.15)	-0.06	PA ≤ 2	PA-	208 (28.9)/219 (29.0)
Gender (G)	0.53 (0.2, 0.87)	0.04	G = 1	F	336 (46.0)/366 (47.7)
Fear of COVID-19 (F)	0.08 (0.03, 0.15)	0.03	F ≥ 6	F+	108 (15.0)/118 (15.5)
Corona news consumption (N)	0.28 (0.1, 0.51)	0.03	N ≥ 3	N+	68 (9.6)/71 (9.5)

The effect of resilience on the severity of depression was significantly mediated by perceived stress. Resilience was negatively associated with perceived stress (*β* = −0.381, *p* < 0.001), which in turn was positively associated with the severity of depression (*β* = 0.377, *p* < 0.001). The reverse pathways, with the severity of depression as a mediator of the effects of resilience on stress, were statistically supported as well, suggesting bi-directional influences.

### 3.3. Subgroup-dependent longitudinal changes in severity of depression

**Figure 4:**
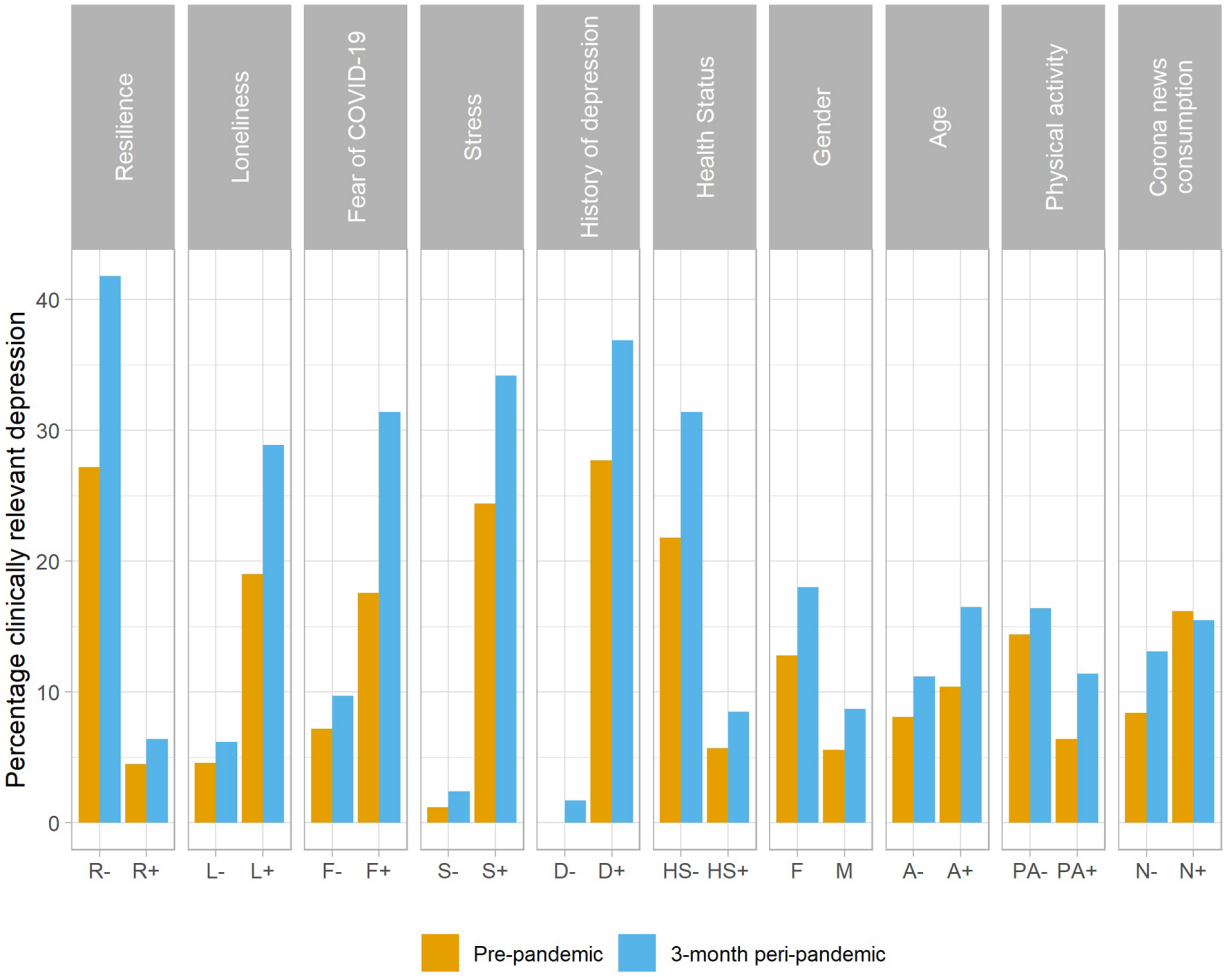
Prevalence (in %) of clinically relevant depression at pre- and 3-month peri-pandemic timepoints for dichotomized variables of the path model of depression severity. Abbreviations and subgrouping criteria are given in Table 2.

Pre-pandemic to 3-month peri-pandemic clinically relevant changes in severity of depression markedly differed between several subgroups stratified by risk factors of depression (Table 2 and Figure 4). In particular, individuals with low resilience (pre-pandemic: 27.2% with mild to severe depression/3-months peri-pandemic: 41.8%), perceived loneliness (19.0%/28.9%), fear of COVID-19 (17.6%/31.4%), high stress (24.4%/34.2%), a positive history of depression (27.7%/36.9%), and low health status (21.8%/31.4%) showed the most severe pre- to 3-month peri-pandemic changes in the prevalence of clinically relevant depression. These six factors were subsequently selected as risk factors of depression.

**Figure 5:**
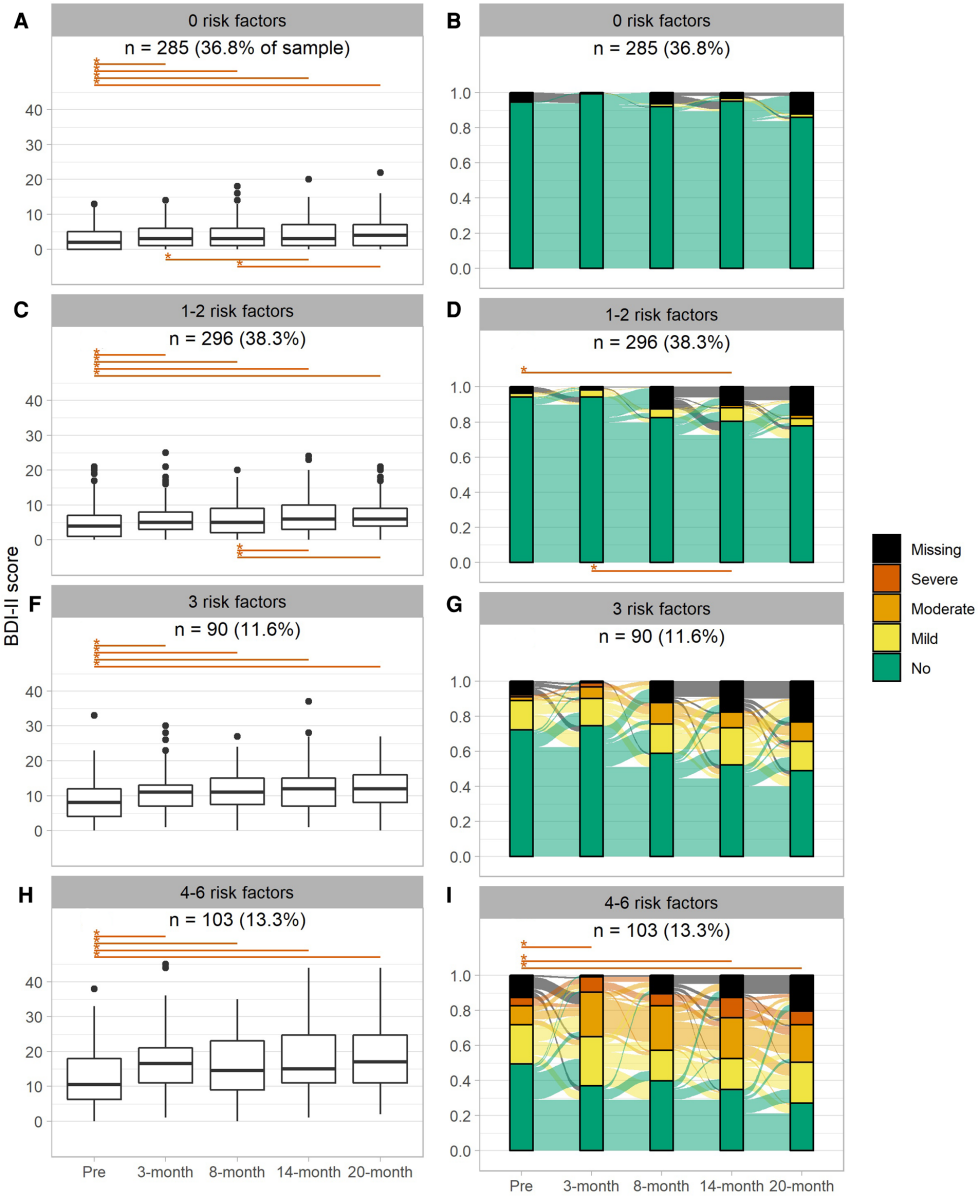
Boxplots (**A,C,E,G**) and alluvial diagrams (**B,D,F,H**) for pre-pandemic and 3-, 8-,14-, 20-months peri-pandemic assessments by subgroup defined by the number of risk factors of depression. Asterisks and lines indicate significant (*p* < 0.00139, Bonferroni corrected) differences between timepoints (red: deterioration, blue: improvement). Tests in the alluvial diagram are based on categorized data as shown in the plot.

Stratification of the longitudinal data on severity of depression by the number of risk factors and determinants of strong early pandemic changes in depression showed subgroup-specific changes over the course of the pandemic. Changes in the severity of depression as a continuous variable (Figures 5 A, C, E, F) and in the categorization of clinically relevant depression (Figures 5 B, D, F, H) are shown over the long-term longitudinal pre- to 20-month peri-pandemic timeframe for individuals with none, one to two, three, and four to six risk factors for depression. Timepoint comparisons of the severity of depression show that, in each group, the most pronounced change is observed between pre- and 3-month peri-pandemic data. For clinically relevant categories of mild to severe depression only individuals with more than three risk factors (Figure 5H) showed a significant sustained increase in depression from pre- to the 20-month peri-pandemic timepoint. Moreover, individuals with one to two risk factors showed a significant increase in clinically relevant depression between 3-month and 14-month peri-pandemic timepoints. However, while the degree of missingness of data was relatively low, missingness generally increased over time, with highest missingness noted for the 20-month peri-pandemic timepoint. To explore which factors are associated with missingness of depression at specific peri-pandemic timepoints, we additionally performed logistic regressions with missingness as dependent variable and clinically relevant depression (yes, no) at the prior timepoint, global cognitive performance (pre-pandemic, based on the CERAD neuropsychological battery sum score (32)) as well as age, gender and years of education as covariates. In particular at later timepoints, clinically relevant depression as well as cognitive performance showed associations with missingness of data, whereas age showed associations at 8-month peri-pandemic. However, effects were heterogenous between timepoints and subgroups (stratified by cumulative risk factors) regarding significance and strength of associations (see Supplementary Tables S5–S7).

For subgroups stratified based on resilience and one additional single risk factor of increased severity of depression, a similar longitudinal peri-pandemic stability and consistency of proportions of mild to severe depression from the 3-month to the 20-month peri-pandemic timeframe was observed (see Supplementary Figures S1–10).

## 4. Discussion

During the COVID-19 pandemic, only specific subgroups of older members of the longitudinally assessed and relatively large TREND cohort showed a substantial increase in clinically relevant depression. These subgroups are characterized by low resilience, loneliness, fear of COVID-19, high level of perceived stress, a positive history of depression, and low general health, and the cumulative number of these risk factors of depression. Subgroup-specific longitudinal changes in the severity of depression were largely observed early in the pandemic, from pre-pandemic to 3-month peri-pandemic timepoints and were largely sustained over 20 months during the COVID-19 pandemic. However, individuals showed large heterogeneity in the flow between categories of the severity of depression between peri-pandemic timepoints, which might reflect the individual constellations and non-linear developments of psychosocial factors and circumstances over the course of the COVID-19 pandemic.

Evidence of a general increase in depressive symptoms due to the COVID-19 pandemic in older populations is inconsistent. Some studies showed no or only a modest increase of depression early in the pandemic in Germany (5, 11, 33), and mental disorders were rather observed in younger individuals and in association with occupational and financial strains (5, 34). A longitudinal study based on 1-year peri-pandemic data of older individuals from the UK showed that mental health outcomes including depression and anxiety continually deteriorate from pre-pandemic to ∼3-month and ∼8-month peri-pandemic timepoints (35). Consistent with our findings, loneliness and quality of life indicating well-being, but not gender or socioeconomic status, substantially affected longitudinal changes in depression from pre- to peri-pandemic phases (35). However, presence of four or more depressive symptoms rather than the severity of depression had been used in that study as an outcome, and quality of life did not only concern health-related aspects but well-being in general, likely limiting the clinical relevance and comparability to the present study.

Data from the SHARE study, taken across 11 European countries without systematic within-country differences, showed an unprecedented decline of the probability of reporting depressive symptoms in older individuals from pre-pandemic to ∼3-months peri-pandemic (May-August 2020) (36). It was noted, that at the time of assessment, most lockdowns had been lifted and fewer and milder restrictions in the summer of 2020 may have relieved many older individuals, thereby reducing the reporting of feelings of depression. Also, in the overall group, older individuals may have been highly resilient in the face of an unparalleled health crisis (33, 37, 38). In the present study, > 80% of older individuals had normal to high resilience, which was the strongest protective factor against increases in the severity of depression due to the pandemic. In addition to resilience, multiple factors including loneliness, fear of COVID-19, perceived stress, history of depression, and low general health are partly associated with one another, and play a role for defining subgroups at risk of severe and clinically relevant increases in depression early in the pandemic. In particular, individuals with four or more risk factors showed most pronounced and sustained increases in clinically relevant depression up to the 20-month peri-pandemic timepoint.

At an individual level, constellations of risk and protective factors might have changed dynamically over time, possibly reflecting negative or positive adaptation to personal and pandemic-related circumstances. Interestingly, all subgroup stratifications showed an initial early pandemic increase in the severity of depression to varying degree that was thereafter largely sustained until the 20-month peri-pandemic timepoint. Thus, the early pandemic phase may have been decisive for the longer-term pandemic-related severity of depression and clinical depression. Despite the temporal peri-pandemic stability on the (sub)group level, profound flow between clinically relevant and non-relevant categories of depression between peri-pandemic timepoints was observed. This may reflect both the aforementioned positive or negative adaptation strategies as well as the complexity of the pandemic situation with its highly non-linear dynamics and many unforeseeable positive (e.g., vaccination, SARS-COV-2 variants causing milder symptoms) and negative developments (e.g., social restrictions, dynamics of incidence rates, increased permissibility of SARS-COV-2 variants).

While perceived stress showed a mediating effect on the association between resilience and the severity of depression, the reverse pathways with the severity of depression as a mediator of the effects of resilience on stress were statistically supported as well. Based on longitudinal pre-pandemic data similar bi-directional influences have been reported for the relationship between perceived isolation as a mediator of the relationship between social disconnectedness and symptoms of depression among older individuals (7) . Our study showed direct associations between loneliness and the severity of depression of similar effect size (*β*=.16) based on cross-sectional 3-month peri-pandemic data. Thus, both stress and perceived isolation may be interrelated with symptoms of depression. Similar to the severity of depression, levels of perceived stress were sustained in many individuals throughout the pandemic, and the stress-related biological long-term effects of the pandemic on the incidence of a variety of diseases require further investigation. In this context, pre-pandemic health status, changes in physical activity as well as individual coping strategies, social networks and various other psychosocial aspects will be important to consider (9, 39, 40).

While individuals with four or more risk factors of depression (13.3% of the sample) may constitute small percentages of older individuals, the absolute number of individuals severely affected by long-term depression is likely to be high given the population-wide and global scale of the COVID-19 pandemic. Also, recent evidence shows that depression and low resilience are also risk factors for long- and post-COVID symptoms (41, 42). While few SARS-CoV2 infections were reported by TREND study participants early in the pandemic until end of 2021, these risk factors have to be considered for evaluating the direct and long-term effects of SARS-CoV2 infections. The COVID-19 pandemic and other worrisome crises globally continue to affect livelihoods and mental and physical health. Individual constellations of risk factors of mental health as well as protective factors and individual coping strategies in times of crises requires further research (43). Both sudden, unanticipated events such as the start of the COVID-19 pandemic as well as long endurance of crises may have to be considered in models of the (subgroup-dependent) temporal dynamics of depression. Additional long-term studies of the incidence of clinical manifestations of depression in vulnerable subgroups and studies and implementations of actionable preventive measures are required (44).

Our study has several strengths including the long-term prospective and comprehensive collection, in a well characterized cohort with good adherence, of data on relevant psychosocial, health- and pandemic-related factors long before as well as early and repeatedly (6-monthly) during the COVID-19 pandemic. Moreover, apart from the effects of single risk factors on changes of depression during the COVID-19 pandemic, the present study also investigated subgroups stratified by the cumulative load of risk factors regarding longitudinal changes of depression.

The following limitations of the study have to be considered as well. 1) The TREND study cohort has been partly enriched with individuals with, for instance, a history of depression which might reduce generalizability of the findings. In comparison, the population-based German National Cohort (NAKO) study showed a higher pre-pandemic prevalence of moderate to severe depression (6.4%) compared to the TREND cohort (3.2%) while the changes from pre- to early pandemic were similar between the cohorts (5). 2) While potential risk factors for depression were pre-selected from the available data and investigated for associations in the path model of the severity of depression, several important aspects were not investigated including other coping strategies, individual mobility, type of participation (online/postal) and social support. 3) Despite good adherence, with <20% of data missing for single timepoints, some of the missingness might not have occurred at random. Our additional analysis of missingness of data revealed that indeed, depending on timepoint and subgroup, age, cognitive deficits and clinically relevant depression may have contributed to the missingness (mostly due to non-response in postal/online surveys) at subsequent timepoints. Thus, the longitudinal data may be biased by non-response and data missing not at random so that depression, and increases thereof, might be partly underestimated. Future studies should consider these factors for improvement of retention and non-response rates, and for developing specific follow-up strategies for older, cognitively impaired and/or depressed individuals. 4) The severity of depression was only assessed using a self-report questionnaire and clinical diagnoses of depression were not available for the peri-pandemic timepoints. Notably, most of the self-report questionnaires on symptoms of depression were conceptualized and validated before the COVID-19 pandemic. Pandemic-related restrictions of everyday life that may have reduced social contacts, self-efficacy, mobility and possibly changes in eating and sleeping habits, could be misinterpreted as symptoms of depression. Lower levels and smaller increments of the severity of depression or the mere number of (mild) depressive symptoms, should be interpreted with caution. Therefore, the present study also investigated longitudinal changes of clinically more relevant levels of depression, i.e., prevalence of mild to severe depression, with inherent clinical relevance. While restrictive measures changed over time throughout the pandemic, the peri-pandemic sustain of pre- to 3-month peri-pandemic changes in depression suggest that these effects were not merely due to BDI-II questionnaire items that may be construed as nonspecific for depression in the context of the pandemic. 5) The cumulative load of risk factors accounts for the heterogeneity of constellations of risk factors of depression that might be relevant for different groups of individuals. Since we did not weight single risk factors (e.g., based on the strength of association with the severity of depression), we however neglected the (overall) amount of risk associated to individual risk factors. Moreover, we did not investigate whether specific constellations of risk factors were associated with a higher risk of longitudinal increases in depression than others, or whether specific clusters of risk factors were more frequent than others.

In conclusion, the present study showed that only older individuals with low resilience, loneliness, fear of COVID-19, perceived stress, a positive history of depression, and low general health showed a clinically relevant increase in depression during the COVID-19 pandemic. Particularly, a significant increase was observed for individuals with four of more of these risk factors of depression. Increases occurred early in the pandemic and were largely sustained over 20 months. The heterogeneity of intra-individual changes of depression severity over time might reflect the complex, individual and non-linear developments of psychosocial factors and circumstances over the course of the COVID-19 pandemic. The temporal dynamics of individual constellations of risk and protective factors of mental health require further research. Targeted risk mitigation of depression should particularly focus on these vulnerable subgroups of older individuals in future crises.

## Data Availability

The raw data supporting the conclusions of this article will be made available by the authors, without undue reservation.
