# Supplementary material for: Longitudinal dynamics of depression in risk groups of older individuals during the COVID-19 pandemic
Source: Front Epidemiol. 2023 Feb 13;3:1093780. doi: 10.3389/fepid.2023.1093780 (PMC10911044; doi:10.3389/fepid.2023.1093780)

*Supplementary Material*

# Supplementary Data

**Supplementary table S1** Number of observations and missing values for the variables used in the comparison of values pre- and peri-pandemic.

|  | Severity of depression | | Health status | | Loneliness | | Physical activity | |
| --- | --- | --- | --- | --- | --- | --- | --- | --- |
|  | Available [n] | Missing [n (%)] | Available [n] | Missing [n (%)] | Available [n] | Missing [n (%)] | Available [n] | Missing [n (%)] |
| Pre | 830 | 47 (5.4%) | 817 | 60 (6.8%) | 808 | 69 (7.9%) | 812 | 65 (7.4%) |
| Coro-Q1 | 768 | 109 (12.4%) | 763 | 114 (13.0%) | 751 | 126 (14.4%) | 760 | 117 (13.3%) |
| Coro-Q2 | 773 | 104 (11.9%) | 773 | 104 (11.9%) | 763 | 114 (13.0%) | 760 | 117 (13.3%) |
| Coro-Q3 | 793 | 84 (9.6%) | 787 | 90 (10.3% | 782 | 95 (10.8%) | 791 | 86 (9.8%) |
| Coro-Q4 | 710 | 167 (19.0%) | 742 | 135 (15.4%) | 739 | 138 (15.7%) | 745 | 132 (15.1%) |

**Supplementary table S2** Variables considered in the comparison of values before and at the beginning of the SARS-Cov-2 pandemic and in the path model.

| Variable | Validated questionnaire (if applicable) | Range / unit |
| --- | --- | --- |
| Severity of depression | Beck’s Depression Inventory II (BDI-II) | 0 – 63 |
| Depression history | ‘Yes’ if self-reported medical lifetime diagnosis of depression at the last onsite TREND visit or BDI-II scores ≥14 in at least one TREND assessment before 01/03/2020  ‘No’ otherwise | 0: no  1: yes |
| Stress | Perceived Stress Scale (PSS) | 0 – 40 |
| Resilience | Brief Resilience Scale (BRS) | 1 – 5 |
| Loneliness | 6-Item De Jong Gierveld Loneliness Scale for Emotional and Social Loneliness | 0 – 6 |
| Fear of COVID-19 |  | 0 – 10 |
| Health status | EQ-5D-5L, visual analog scale | 0 – 100 |
| Physical activity |  | 1: no  2: $<$ 1h per week  3: 1-2h per week  4: 2-4h per week  5: $>$4h per week |
| Corona news consumption |  | 0: no  1: $<$ 1h per day  2: 1-2h per day  3: 2-4h per day  4: $>$4h per day |
| Education |  | Years |
| Gender |  | 0: male  1: female |
| Age |  | Years |

**Supplementary table S3** Pairwise comparisons between the five timepoints for changes in severity of depression, health status, loneliness and physical activity.

| Timepoint comparison | | Severity of depression | Health status | Loneliness | Physical activity |
| --- | --- | --- | --- | --- | --- |
| pre / 3-month | p | 0.00000 | 0.07521 | 0.00000 | 0.00001 |
|  | n | 725 | 709 | 694 | 707 |
|  | Intra-individual differences [mean (sd)] | 1.4 (4.1) | -2.0 (16.3) | 0.3 (1.4) | -0.2 (1.1) |
| pre / 8-month | p | 0.00000 | 0.00000 | 0.00000 | 0.00018 |
|  | n | 734 | 724 | 707 | 710 |
|  | Intra-individual differences [mean (sd)] | 1.7 (4.4) | -3.0 (15.6) | 0.4 (1.4) | -0.2 (1.1) |
| pre / 14-month | p | 0.00000 | 0.00000 | 0.00000 | 0.00000 |
|  | n | 758 | 740 | 730 | 738 |
|  | Intra-individual differences [mean (sd)] | 2.3 (5.0) | -3.9 (16.4) | 0.5 (1.4) | -0.2 (1.2) |
| pre / 20-month | p | 0.00000 | 0.00000 | 0.00001 | 0.00083 |
|  | n | 675 | 697 | 690 | 697 |
|  | Intra-individual differences [mean (sd)] | 2.4 (4.8) | -5.2 (16.6) | 0.2 (1.3) | -0.1 (1.1) |
| 3-month / 8-month | p | 0.52128 | 0.05482 | 0.00905 | 0.83482 |
|  | n | 694 | 686 | 669 | 674 |
|  | Intra-individual differences [mean (sd)] | 0.1 (3.9) | -0.6 (16.8) | 0.1 (1.2) | 0.0 (1.1) |
| 8-month / 14-month | p | 0.00000 | 0.00025 | 0.01487 | 0.10911 |
|  | n | 730 | 722 | 712 | 715 |
|  | Intra-individual differences [mean (sd)] | 0.7 (3.9) | -1.8 (15.8) | 0.1 (1.2) | -0.1 (1.1) |
| 14-month / 20-month | p | 0.26979 | 0.04373 | 0.00000 | 0.00824 |
|  | n | 683 | 713 | 702 | 717 |
|  | Intra-individual differences [mean (sd)] | 0.1 (3.9) | -1.2 (16.1) | -0.3 (1.2) | 0.1 (1.1) |
| 3-month / 14-month | p | 0.00000 | 0.00001 | 0.00008 | 0.02731 |
|  | n | 702 | 691 | 680 | 692 |
|  | Intra-individual differences [mean (sd)] | 0.8 (4.1) | -2.4 (17.6) | 0.2 (1.2) | -0.1 (1.1) |
| 8-month / 20-month | p | 0.00000 | 0.00019 | 0.00262 | 0.37834 |
|  | n | 657 | 685 | 675 | 676 |
|  | Intra-individual differences [mean (sd)] | 0.7 (3.9) | -2.4 (16.6) | -0.1 (1.3) | 0.0 (1.1) |

**Supplementary table S4** Pairwise comparisons between the five timepoints for changes in depression categories and health status categories.

| Timepoint comparison | | Depression categories | Health status categories |
| --- | --- | --- | --- |
| pre / 3-month | p | 0.00012 | 0.00208 |
|  | n | 725 | 709 |
|  | Intra-individual differences [mean (sd)] | 0.1 (0.4) | -0.1 (0.9) |
| pre / 8-month | p | 0.00000 | 0.00000 |
|  | n | 734 | 724 |
|  | Intra-individual differences [mean (sd)] | 0.1 (0.5) | -0.2 (0.9) |
| pre / 14-month | p | 0.00000 | 0.00000 |
|  | n | 758 | 740 |
|  | Intra-individual differences [mean (sd)] | 0.1 (0.5) | -0.2 (0.9) |
| pre / 20-month | p | 0.00000 | 0.00000 |
|  | n | 675 | 697 |
|  | Intra-individual differences [mean (sd)] | 0.1 (0.5) | -0.2 (0.9) |
| 3-month / 8-month | p | 0.07624 | 0.03903 |
|  | n | 694 | 686 |
|  | Intra-individual differences [mean (sd)] | 0.0 (0.4) | -0.1 (0.9) |
| 8-month / 14-month | p | 0.03209 | 0.30462 |
|  | n | 730 | 722 |
|  | Intra-individual differences [mean (sd)] | 0 (0.4) | 0 (0.9) |
| 14-month / 20-month | p | 0.97653 | 0.27465 |
|  | n | 683 | 713 |
|  | Intra-individual differences [mean (sd)] | 0 (0.4) | 0 (0.9) |
| 3-month / 14-month | p | 0.00039 | 0.00232 |
|  | n | 702 | 691 |
|  | Intra-individual differences [mean (sd)] | 0.1 (0.4) | -0.1 (0.9) |
| 8-month / 20-month | p | 0.02815 | 0.16020 |
|  | n | 657 | 685 |
|  | Intra-individual differences [mean (sd)] | 0.0 (0.4) | -0.1 (0.9) |

**
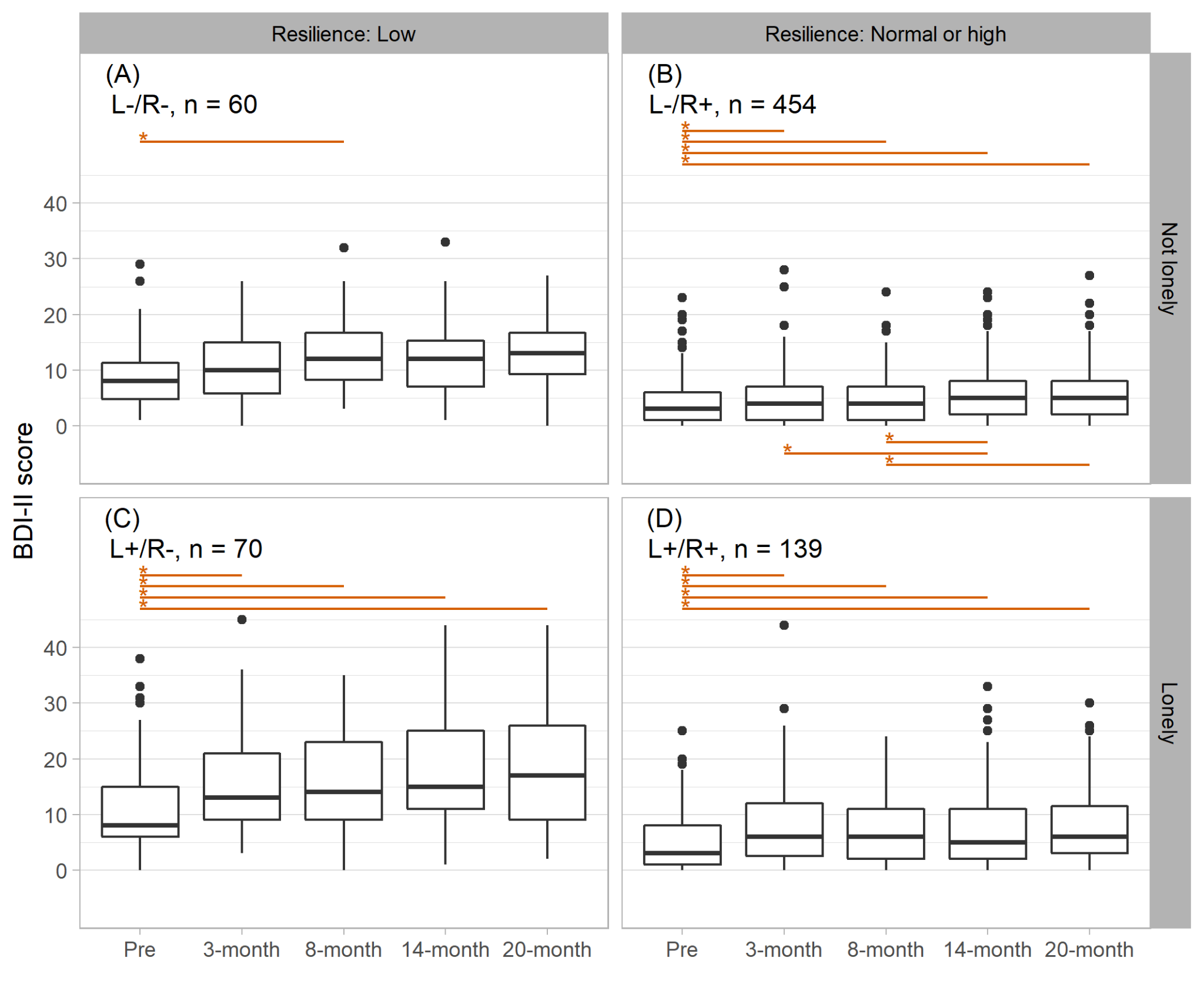
 Supplementary figure S1** Longitudinal change of severity of depression in four subgroups defined by resilience and loneliness at 3-month peri-pandemic. Asterisks and lines indicate significant (p < 0.00139, Bonferroni corrected) differences between timepoints (red: deterioration, blue: improvement).

**
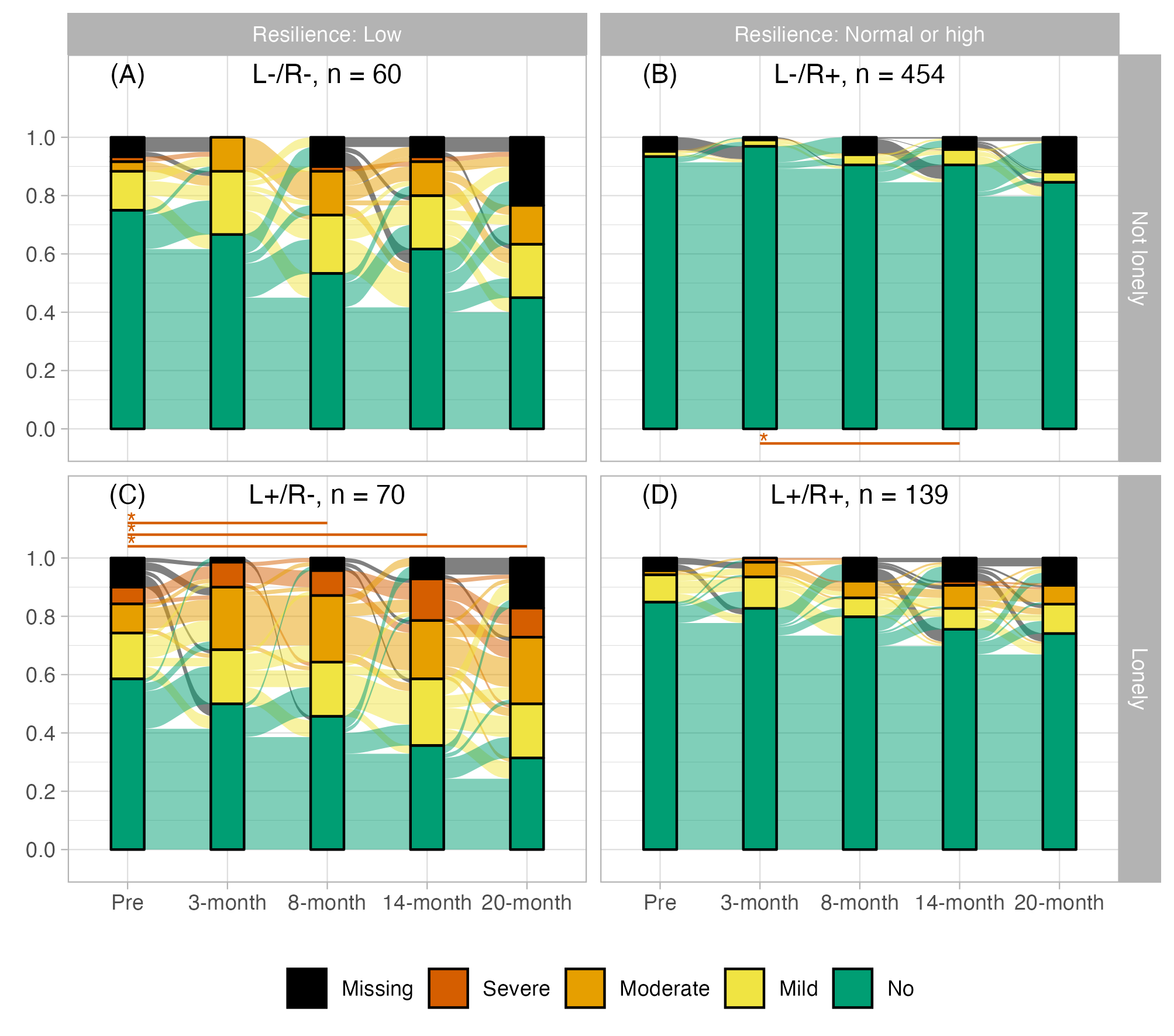
Supplementary figure S2** Relative frequencies of depression categories in subgroups defined by resilience and loneliness at 3-month peri-pandemic and individual changes between categories. Asterisks and lines indicate significant (p < 0.00139, Bonferroni corrected) differences between timepoints (red: deterioration, blue: improvement). Tests are based on categorized data as shown in the plot.


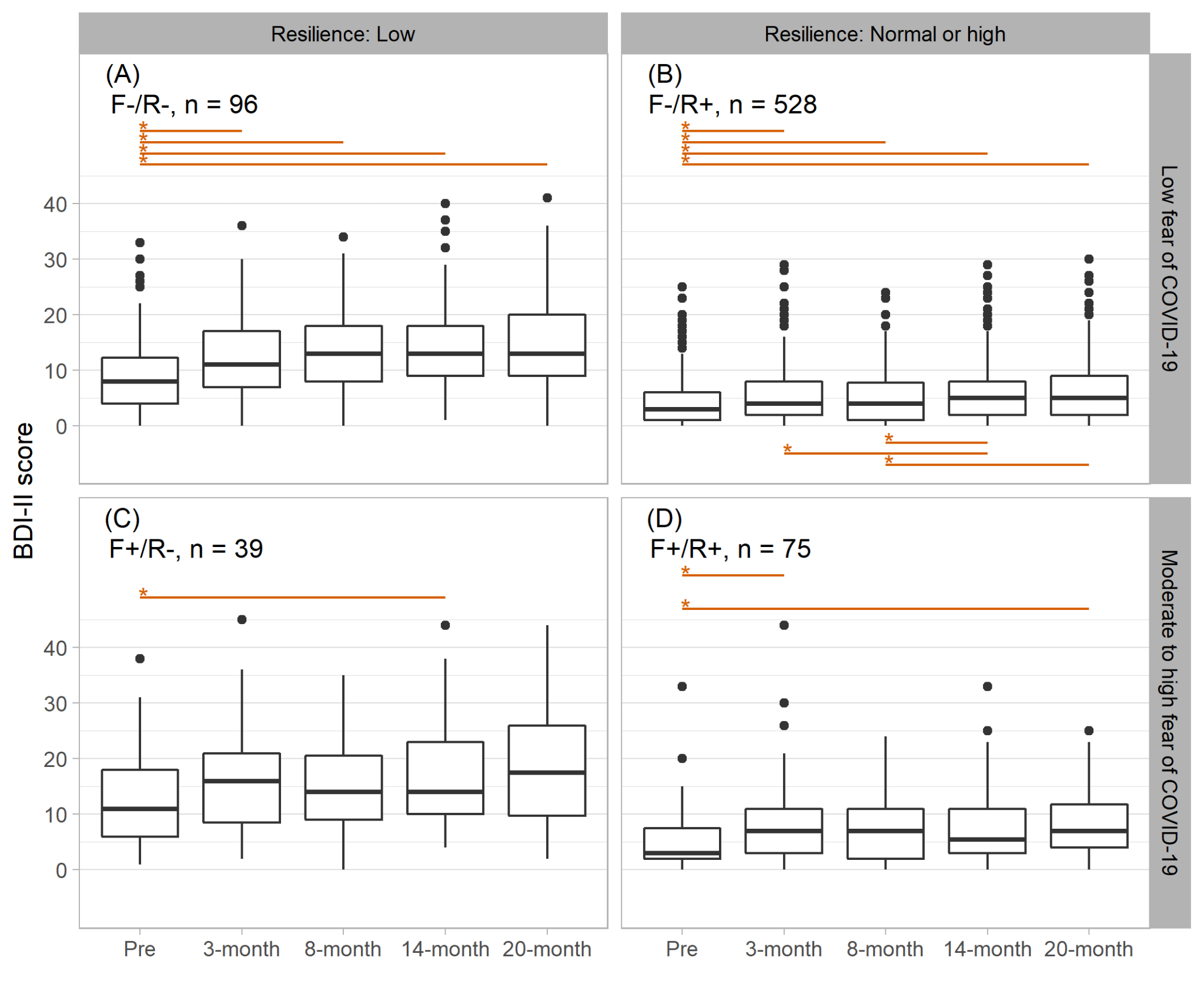


**Supplementary figure S3** Longitudinal change of severity of depression in four subgroups defined by resilience and fear of COVID-19 at 3-month peri-pandemic. Asterisks and lines indicate significant (p < 0.00139, Bonferroni corrected) differences between timepoints (red: deterioration, blue: improvement).

**
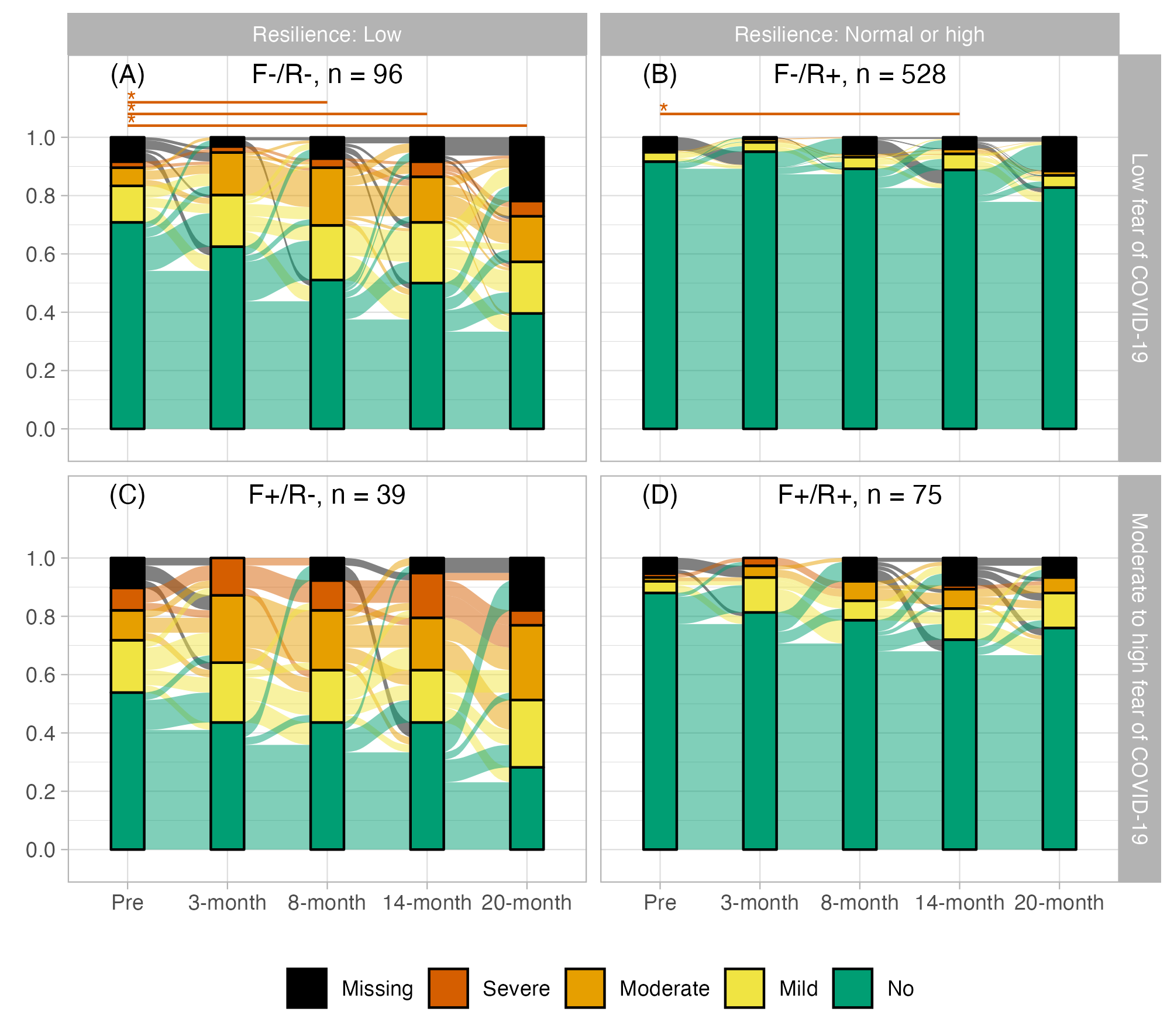
Supplementary figure S4** Relative frequencies of depression categories in subgroups defined by resilience and fear of COVID-19 at 3-month peri-pandemic and individual changes between categories. Asterisks and lines indicate significant (p < 0.00139, Bonferroni corrected) differences between timepoints (red: deterioration, blue: improvement). Tests are based on categorized data as shown in the plot.


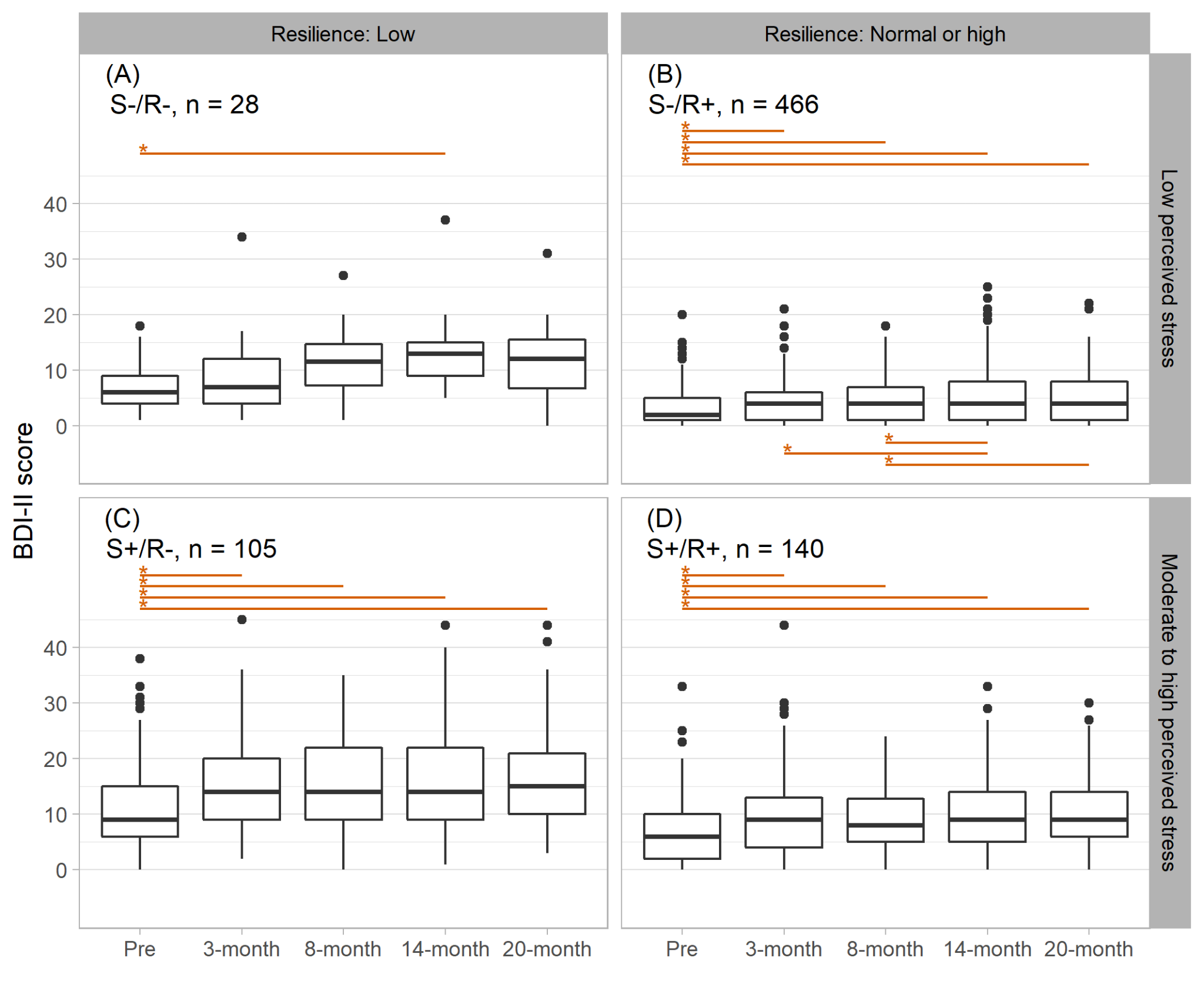


**Supplementary figure S5** Longitudinal change of severity of depression in four subgroups defined by resilience and perceived stress at 3-month peri-pandemic. Asterisks and lines indicate significant (p < 0.00139, Bonferroni corrected) differences between timepoints (red: deterioration, blue: improvement).

**
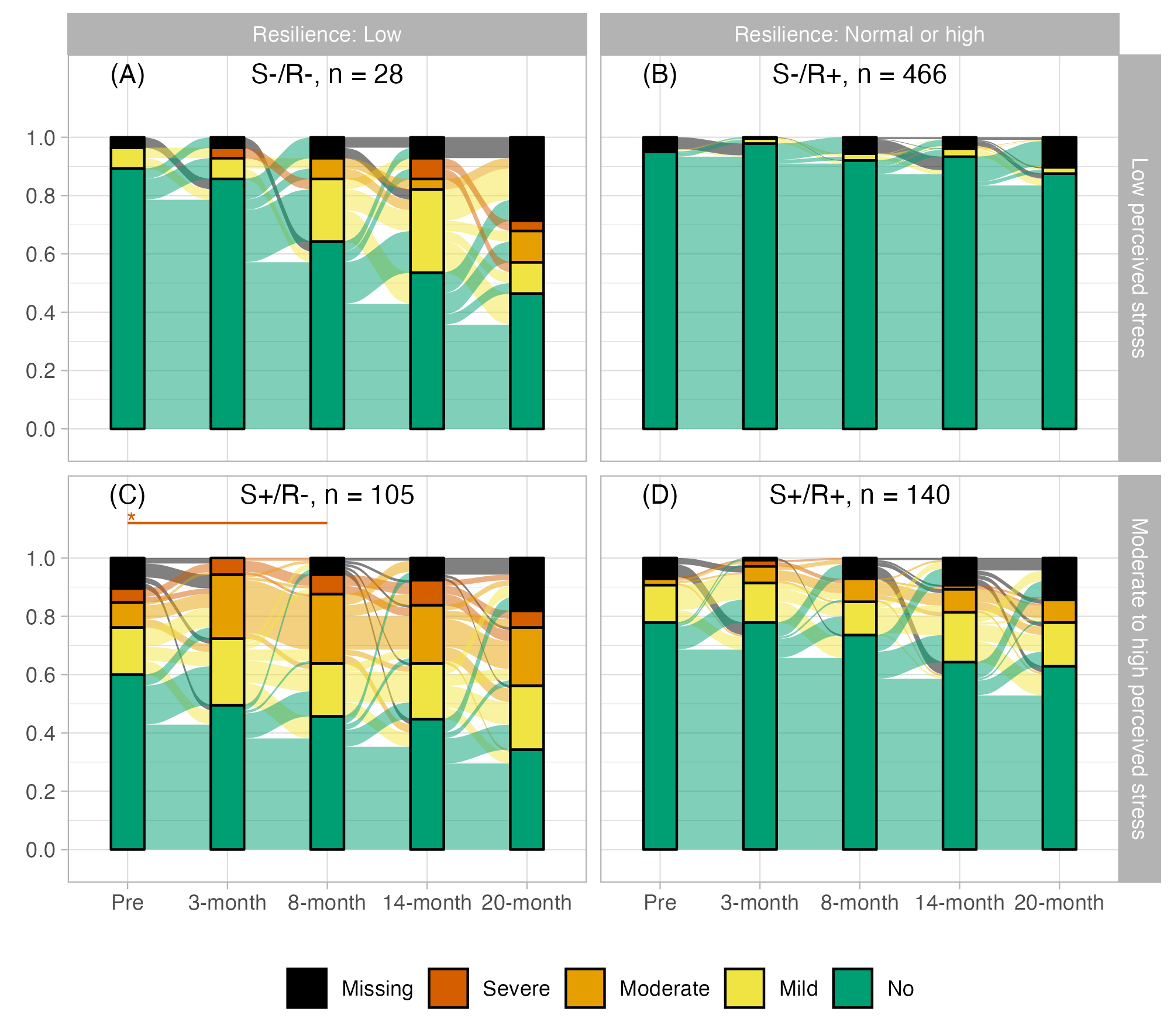
Supplementary figure S6** Relative frequencies of depression categories in subgroups defined by resilience and perceived stress at 3-month peri-pandemic and individual changes between categories. Asterisks and lines indicate significant (p < 0.00139, Bonferroni corrected) differences between timepoints (red: deterioration, blue: improvement). Tests are based on categorized data as shown in the plot.


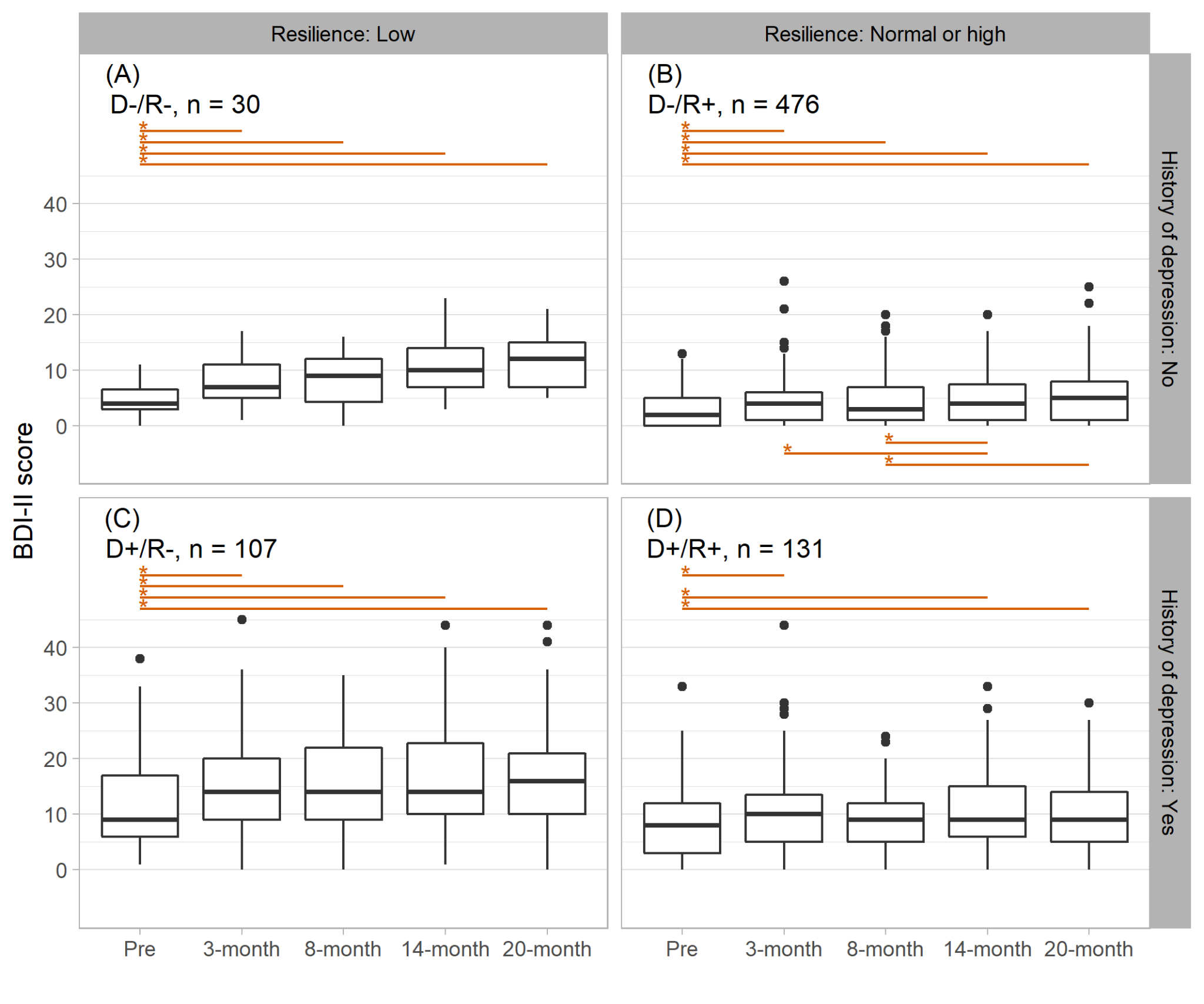


**Supplementary figure S7** Longitudinal change of severity of depression in four subgroups defined by resilience and history of depression at 3-month peri-pandemic. Asterisks and lines indicate significant (p < 0.00139, Bonferroni corrected) differences between timepoints (red: deterioration, blue: improvement).

**
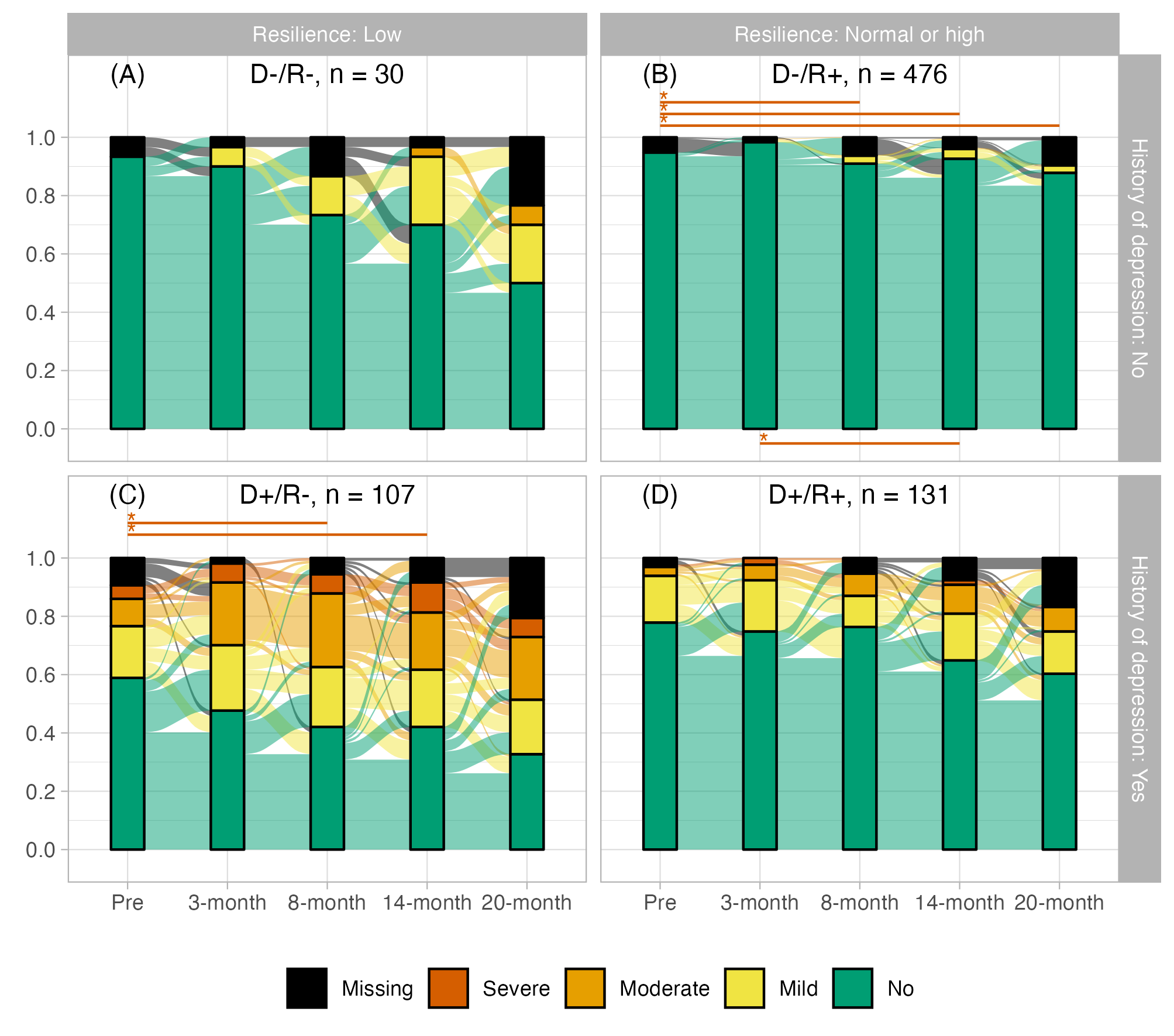
Supplementary figure S8** Relative frequencies of depression categories in subgroups defined by resilience and history of depression at 3-month peri-pandemic and individual changes between categories. Asterisks and lines indicate significant (p < 0.00139, Bonferroni corrected) differences between timepoints (red: deterioration, blue: improvement). Tests are based on categorized data as shown in the plot.


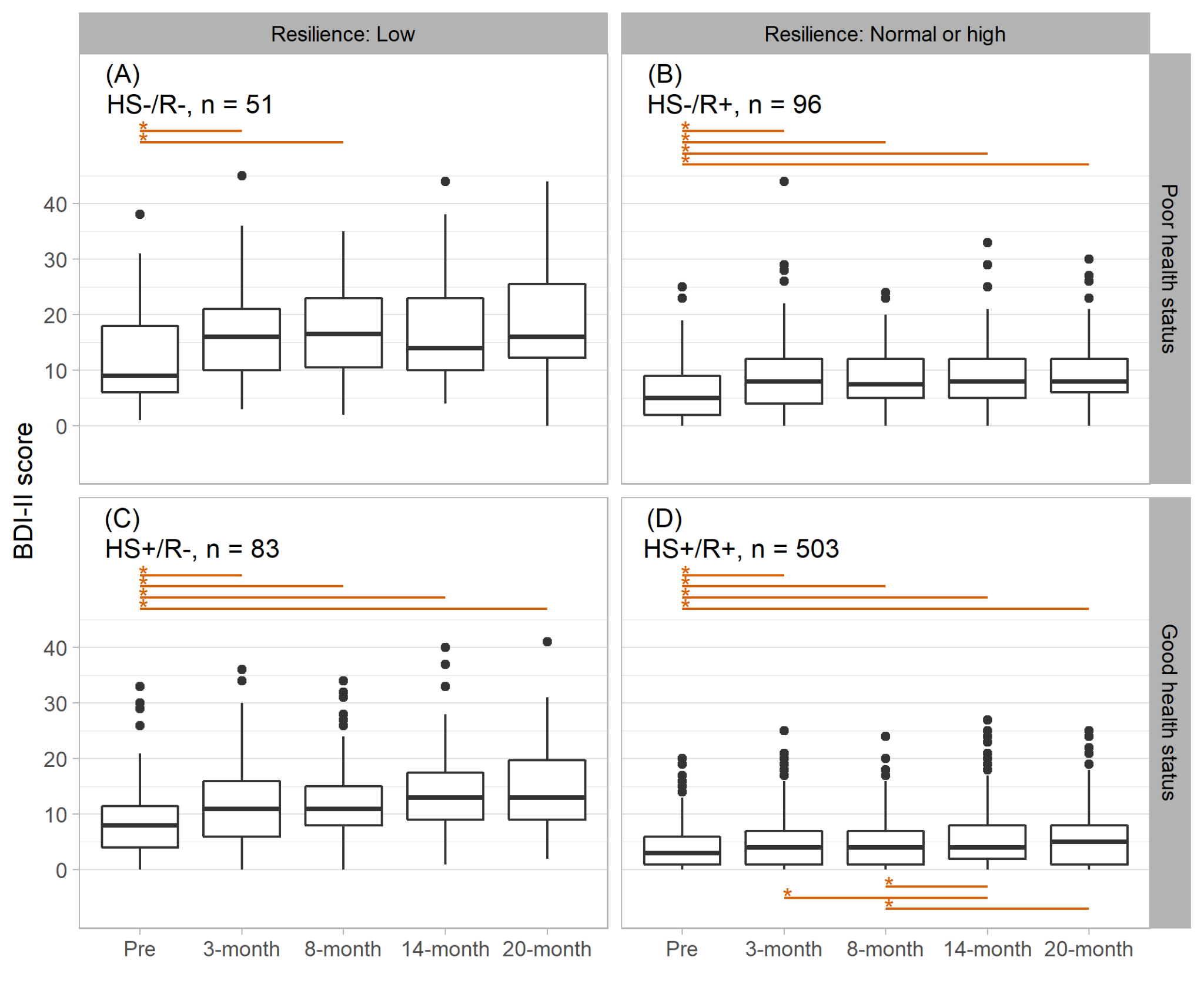


**Supplementary figure S9** Longitudinal change of severity of depression in four subgroups defined by resilience and health status at 3-month peri-pandemic. Asterisks and lines indicate significant (p < 0.00139, Bonferroni corrected) differences between timepoints (red: deterioration, blue: improvement).

**
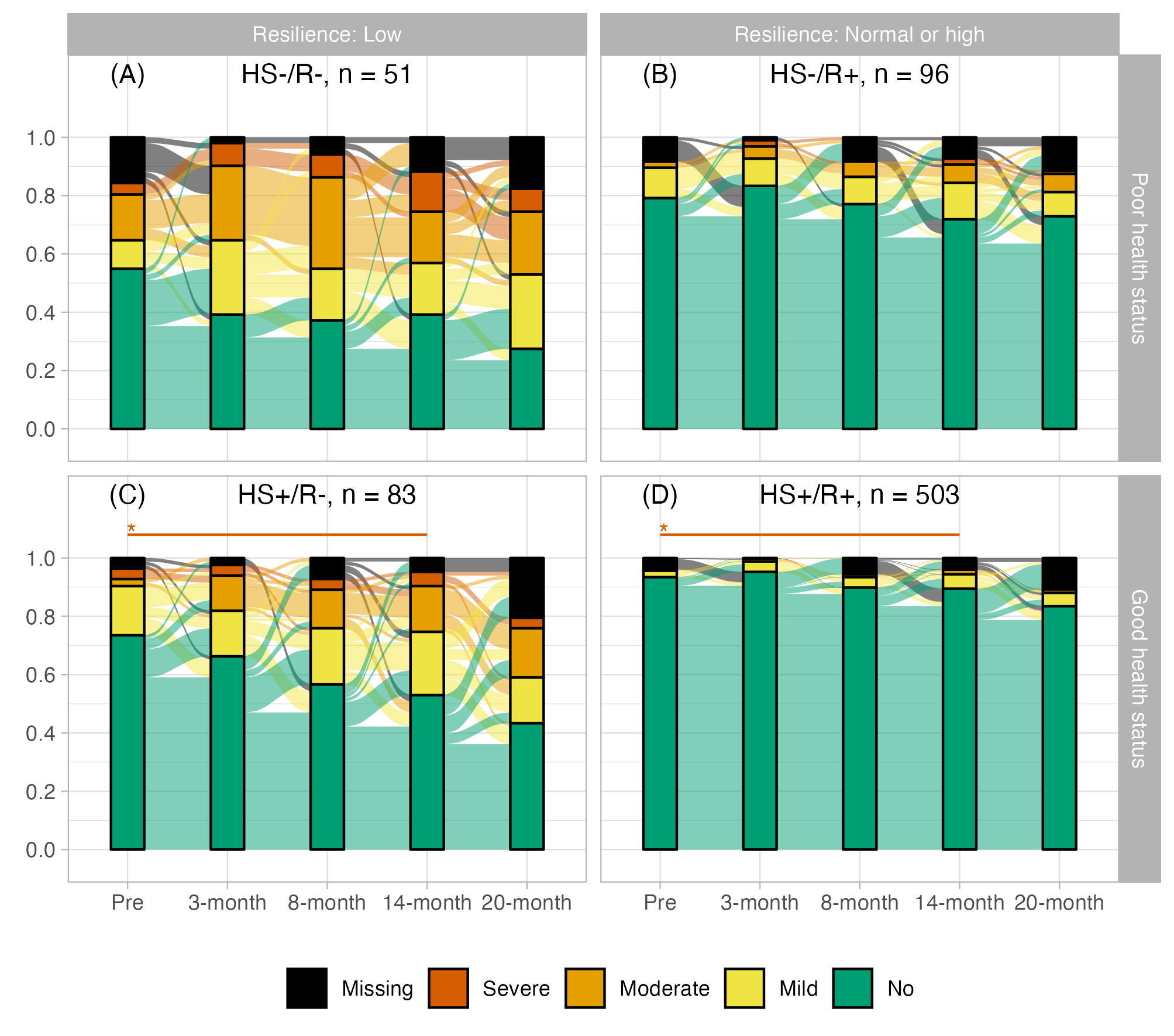
Supplementary figure S10** Relative frequencies of depression categories in subgroups defined by resilience and health status at 3-month peri-pandemic and individual changes between categories. Asterisks and lines indicate significant (p < 0.00139, Bonferroni corrected) differences between timepoints (red: deterioration, blue: improvement). Tests are based on categorized data as shown in the plot.

**Supplementary table S5 Missingness at Coro-Q2 (8-month peri-pandemic)** Results of logistic regression models with missingness of depression at Coro-Q2 as dependent variable and clinically relevant depression (yes, no) at Coro-Q1, global cognitive performance (pre-pandemic, based on the CERAD neuropsychological battery sum score) as well as age, gender and years of education as covariates.

|  | Total sample | | Subgroup with 0 risk factors | | Subgroup with 1-2 risk factors | | Subgroup with 3 risk factors | | Subgroup with 4-6 risk factors | |
| --- | --- | --- | --- | --- | --- | --- | --- | --- | --- | --- |
| Covariates | $\beta$ | p | $\beta$ | p | $\beta$ | p | $\beta$ | p | $\beta$ | p |
| (Intercept) | -3.35 | 0.159 | -5.19 | 0.320 | -4.74 | 0.177 | -8.92 | 0.141 | 5.21 | 0.497 |
| Clinically relevant depression | 0.39 | 0.240 | -12.84 | 0.993 | -0.98 | 0.363 | 0.81 | 0.275 | 1.16 | 0.185 |
| CERAD sum score | -0.01 | 0.372 | 0.01 | 0.731 | -0.02 | 0.366 | 0.04 | 0.361 | -0.05 | 0.370 |
| Age | 0.05 | **0.032** | 0.03 | 0.519 | 0.08 | **0.014** | 0.07 | 0.196 | -0.04 | 0.520 |
| Gender | -0.09 | 0.735 | 0.25 | 0.646 | -0.47 | 0.252 | 1.01 | 0.258 | -1.09 | 0.149 |
| Years of education | -0.07 | 0.157 | -0.05 | 0.625 | -0.10 | 0.240 | -0.17 | 0.253 | -0.05 | 0.687 |

**Supplementary table S6. Missingness at Coro-Q3 (14-month peri-pandemic)** Results of logistic regression models with missingness of depression at Coro-Q3 as dependent variable and clinically relevant depression (yes, no) at Coro-Q2, global cognitive performance (pre-pandemic, based on the CERAD neuropsychological battery sum score) as well as age, gender and years of education as covariates.

|  | Total sample | | Subgroup with 0 risk factors | | Subgroup with 1-2 risk factors | | Subgroup with 3 risk factors | | Subgroup with 4-6 risk factors | |
| --- | --- | --- | --- | --- | --- | --- | --- | --- | --- | --- |
| Covariates | $\beta$ | p | $\beta$ | p | $\beta$ | p | $\beta$ | p | $\beta$ | p |
| (Intercept) | -0.69 | 0.782 | 4.31 | 0.562 | -2.21 | 0.557 | -6.59 | 0.203 | 4.20 | 0.551 |
| Clinically relevant depression | 0.73 | **0.021** | -14.07 | 0.993 | -0.08 | 0.919 | 0.50 | 0.417 | 0.67 | 0.336 |
| CERAD sum score | -0.03 | **0.033** | -0.06 | 0.219 | -0.03 | 0.215 | 0.01 | 0.788 | -0.04 | 0.442 |
| Age | 0.03 | 0.139 | -0.02 | 0.734 | 0.07 | **0.047** | 0.06 | 0.223 | -0.03 | 0.599 |
| Gender | -0.06 | 0.838 | 0.55 | 0.491 | -0.42 | 0.338 | 0.89 | 0.206 | -1.52 | **0.027** |
| Years of education | -0.10 | 0.082 | -0.10 | 0.573 | -0.19 | 0.053 | -0.05 | 0.688 | -0.03 | 0.811 |

**Supplementary table S7. Missingness at Coro-Q4 (20-month peri-pandemic)** Results of logistic regression models with missingness of depression at Coro-Q4 as dependent variable and clinically relevant depression (yes, no) at Coro-Q3, global cognitive performance (pre-pandemic, based on the CERAD neuropsychological battery sum score) as well as age, gender and years of education as covariates.

|  | Total sample | | Subgroup with 0 risk factors | | Subgroup with 1-2 risk factors | | Subgroup with 3 risk factors | | Subgroup with 4-6 risk factors | |
| --- | --- | --- | --- | --- | --- | --- | --- | --- | --- | --- |
| Covariates | $\beta$ | p | $\beta$ | p | $\beta$ | p | $\beta$ | P | $\beta$ | p |
| (Intercept) | -0.82 | 0.668 | -0.45 | 0.906 | -1.83 | 0.546 | -5.14 | 0.263 | 6.81 | 0.223 |
| Clinically relevant depression | 0.47 | 0.054 | -14.71 | 0.988 | 0.12 | 0.822 | 1.08 | 0.051 | 0.02 | 0.973 |
| CERAD sum score | -0.03 | **0.015** | -0.03 | 0.331 | -0.03 | 0.085 | 0.00 | 0.889 | -0.07 | 0.065 |
| Age | 0.03 | 0.095 | 0.01 | 0.697 | 0.05 | 0.110 | 0.06 | 0.181 | -0.02 | 0.735 |
| Gender | 0.08 | 0.701 | -0.05 | 0.908 | 0.31 | 0.374 | -0.08 | 0.884 | -0.32 | 0.582 |
| Years of education | -0.04 | 0.393 | -0.02 | 0.776 | -0.04 | 0.596 | -0.06 | 0.550 | -0.07 | 0.479 |


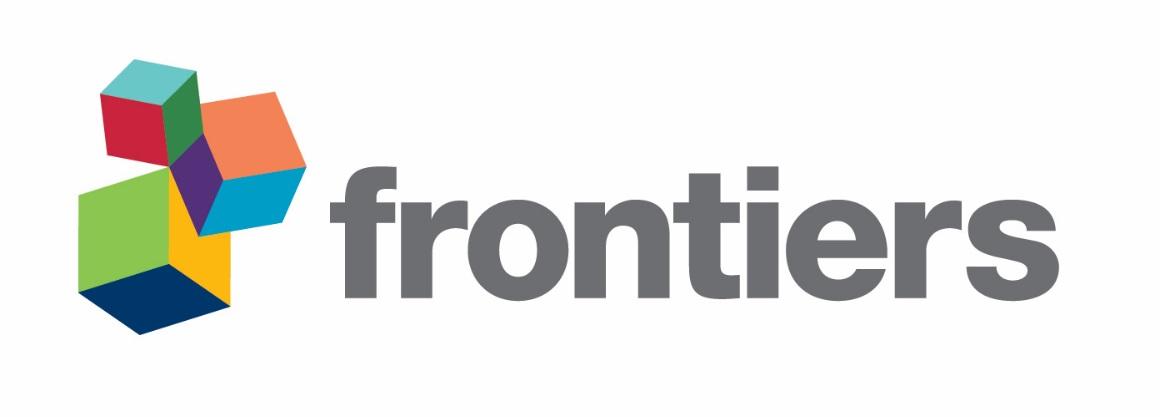

Supplement: Supplementary file 1 [file Datasheet1.docx]
